# Refining the Clinical Pathway for Nasotracheal Intubation: An Updated Decision Making Algorithm

**DOI:** 10.3390/jcm14217746

**Published:** 2025-10-31

**Authors:** Mahesh Desilva, Ramneek Maan, Muhammad Helwany, Shalini S. Shah

**Affiliations:** Department of Anesthesia, Riverside Community Hospital, Riverside, CA 92501, USA

**Keywords:** nasotracheal intubation, orotracheal to nasotracheal intubation conversion, video laryngoscopy, mitigating epistaxis

## Abstract

Nasotracheal Intubation (NTI) is a common route of airway management in many situations. Over the years, numerous techniques and approaches have been described in performing NTI safely and effectively, including many innovative techniques being published in recent years. However, there hasn’t been a summary of the recent approaches to NTI, especially in an easy, clinically applicable decision making format. In fact, the last algorithmic approach to nasal intubation in the literature was published in 2008. This comprehensive review details an updated analysis of NTI techniques along with a new adapted algorithmic outline to approach NTI in a methodical and stepwise manner. There is also an extensive review of techniques to control epistaxis, which is the most commonly encountered complication during NTI. The newly adapted NTI algorithm simplifies the initial approach to three options: Routine Asleep NTI, Awake NTI, and an Initial Orotracheal Intubation (OTI) followed by Conversion to NTI. Older techniques, such as blind NTI, flexible intubation scope guided, “look before you leap” approach and cuff inflation technique, are discussed along with incorporation of newer techniques, such as videolaryngoscope guided, hybrid, alignment approach, and OTI to NTI conversion. Uniquely, this manuscript reviews all published techniques for converting OTI to NTI and categorizes them into two pathways: direct conversion (with glottic visualization) or indirect conversion (without glottic visualization). Furthermore, original animated videos have been created and attached to help elucidate these conversion techniques visually.

## 1. Introduction

Nasotracheal Intubation (NTI) is a common route of airway management in numerous situations, either to facilitate oral and maxillofacial surgery or in a difficult airway scenario [1,2,3,4]. Piepho et al. in 2005 [5] developed a foundational algorithm for approaching NTI. He advocated for a “look before you leap” approach with direct laryngoscopy to assess the potential for a difficult intubation prior to passing the nasal endotracheal tube (NETT) through the nose [5]. Then, in 2008, Backman et al. [6] further adapted the NTI algorithm to include oral intubation as a consideration when an unanticipated grade 3 or 4 difficult Cormack–Lehane view was obtained during laryngoscopy. He also listed several techniques to subsequently convert an orotracheal intubation (OTI) over to a NTI, including a novel approach using an Eschmann bougie [6].

**Figure 1:**
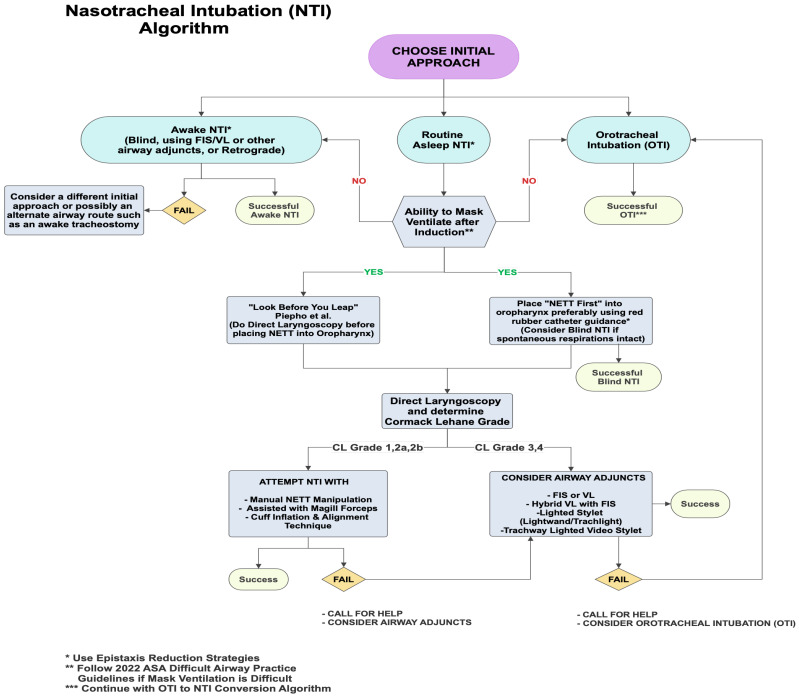
Illustrates a Nasotracheal Intubation (NTI) algorithm including the 3 initial approaches and a decision making tree for the “routine NTI” (RNTI) pathway (modified from Backman S et al. and reproduced with permission) [5,6]. NTI = Nasotracheal Intubation; OTl = Orotracheal Intubation; OETT = Oral Endotracheal Tube; FIS = Flexible intubation Scope; AEC = Airway Exchange Catheter; VL = Videolaryngoscope; NETT = Nasal Endotracheal Tube.

Since the last format of the NTI algorithm in 2008, more airway techniques, advanced airway equipment, and more OTI to NTI conversion techniques have emerged, prompting an update of the Piepho et al. and Backman et al. NTI algorithm [5,6]. This clinical review details an analysis of NTI techniques, old and new, along with an updated clinical decision algorithm to approach NTI in a methodical and stepwise manner (Figure 1). Of note, the NTI algorithm has been updated to incorporate both resource-poor locations around the world without access to modern devices, such as a flexible intubation scope (FIS) or a videolaryngoscope (VL), along with more resource-rich locations.

## 2. Choosing an Initial NTI Approach

One of the main adaptations of the updated NTI algorithm from the prior algorithms is deciding on the initial approach. The most traditionally used is the “routine NTI” (RNTI) asleep approach with induction followed by mask ventilation and direct laryngoscopy (DL), often utilizing Magill forceps [7]. (Video S1) https://m.youtube.com/watch?v=RxWc8JK0_iE (accessed on 1 October 2025). A second NTI approach available for a cooperative patient is an awake NTI with conscious sedation while maintaining spontaneous respiratory effort and intact airway reflexes [8]. Awake NTI is probably a safer approach when difficult ventilation or intubation is anticipated pre-induction and when mouth opening is mechanically limited, such as in patients with temporomandibular joint ankylosis [9]. A third NTI approach that can be considered INITIALLY (versus when a difficult intubation is encountered, as Backman et al. suggested [6]) is an orotracheal intubation (OTI) and a subsequent conversion to NTI. Since securing the airway orally is typically easier and quicker than a NTI, especially with utilization of new devices such as VL, this approach may be popular when a difficult RNTI is anticipated or there is desire to minimize mask ventilation during NTI. In addition, an initial asleep OTI is more comfortable for patients compared to an awake intubation, suitable for uncooperative patients, and can give the clinician additional time to employ further nasal epistaxis reduction measures if desired.

### 2.1. Routine Asleep NTI (RNTI) Approach

One of the critical steps in the RNTI approach is the ability to adequately mask ventilate the patient after induction. Difficult and/or inadequate mask ventilation despite optimization maneuvers is probably best managed by either:

(1) Quickly attempting an OTI.
(2) Placing a supraglottic airway (SGA).
(3) Waking the patient up and attempting an awake NTI.

The ASA 2022 Difficult Airway Practice Guidelines will assist the clinician to manage the patient safely until adequate ventilation and oxygenation are achieved [10].

But if mask ventilation is adequate, as it typically is, then the anesthesia clinician can consider two options:

(1) The “Look before you leap” option advocated by Piepho et al. [5] involves obtaining a glottic view with DL before placing the NETT into the oropharynx. Piepho et al. [5] recommended passing the NETT through the nares only in patients with a grade 1 or 2 Cormack–Lehane (CL) view and using alternate adjunct guided techniques otherwise [5]. Lesser experienced clinicians may find this approach more reassuring.
(2) A more traditional option is placing the NETT into the oropharynx first (“NETT first”) before obtaining a glottic view with DL. The downside to this approach is that the clinician may occasionally encounter a difficult 3/4 CL grade during subsequent DL, making it challenging to advance the NETT situated in the oropharynx through the glottis. Albeit rare, there is also potential for severe epistaxis with initial NETT insertion, which may lead to aspiration of blood and render alternative options, such as FIS-assisted NTI, difficult [11]. Telescoping the NETT with a red rubber Robinson catheter, amongst other epistaxis reduction measures, is encouraged to reduce the possibility of severe epistaxis and help guide the NETT through the nasal pathway [12].

#### 2.1.1. RNTI Scenario with CL Grade 1/2 View

Often in this scenario, with a complete or partial view of the glottis, the clinician will not have difficulty placing the NETT into the trachea, either with manual manipulation or assisted with Magill forceps. Caution has to be used when using Magill forceps to avoid accidental injury to the delicate pharyngeal tissues, uvula, and/or prevent damage to the NETT cuff, which may lead to a cuff leak requiring NTI replacement [13].

A commonly used technique to supplement manual manipulation and potentially avoid the use of Magill forceps is the cuff inflation technique, wherein the cuff of the NETT is inflated with ~10–20 mL of air to raise its tip until it is aligned with the glottis, and then the NETT tip is moved forward to partially enter the glottis followed by deflation of the cuff so that the NETT can be advanced further into the trachea to complete the NTI [14]. The cuff inflation technique is especially useful when thermosoftened NETTs are utilized, which causes loss of the anterior curvature of the NETTs, directing them posteriorly towards the esophagus [15]. A modification of the cuff inflation technique is the alignment approach, where, in addition to cuff inflation, purposeful limitation of the glottic view to a CL grade 2 during laryngoscopy is undertaken to reduce the chances of the abutment of the NETT on the anterior tracheal wall [16].

The 90-degree counterclockwise rotation and Seldinger techniques using a bougie can be helpful to facilitate NETT advancement past the glottis if the NETT becomes caught up on laryngeal structures or if resistance is encountered due to abutment against the tracheal wall [17,18]. In addition, the Chula formula [9 + (body height/10) cm] can help estimate the proper length for securing the NETT in the trachea at the right external naris [19].

#### 2.1.2. RNTI Scenario with CL Grade 3/4 View

When confronted with a difficult CL grade 3/4 view during RNTI, a clinician can quickly try simple maneuvers to improve the glottic view, such as changing the DL blade, optimizing patient position, and external manipulation of the larynx. Also, if the patient desaturates while the NETT is already in the oropharynx, the NETT can be used to ventilate and oxygenate the patient while closing the mouth and opposite nare, similar to ventilating through a nasal airway [20].

If simple maneuvers do not improve the DL glottic view to CL 1/2, then adjunct devices will likely be needed to assist with the NTI. The most commonly used adjunct devices during a RNTI scenario with a CL 3/4 view are probably a VL or FIS, which are especially useful when patients also have a limited mouth opening, making DL and RNTI difficult. However, in patients with severely limited mouth opening, FIS, as opposed to VL, is likely the more suitable option as the size of the VL blade can make its insertion into the oral cavity particularly challenging in the presence of trismus. 

When FIS is employed for NTI, the use of a “NETT first” option is recommended for a higher success rate with minimal attempts [21,22]. Furthermore, pre-insertion of the NETT to a depth of 14 cm prior to insertion of the FIS typically results in excellent visualization of the glottis to intubate the trachea easily and quickly [23].

Many VLs have been used successfully to achieve NTI, including non-channeled VLs, such as Glidescope, CMAC, and McGrath, or channeled VLs, such as Airtraq, King Vision, and Pentax Airway Scope [24]. VLs have been consistently shown to provide a better view of the laryngeal structures, facilitating the ease and speed of NTI [25,26,27,28,29]. In fact, many clinicians in resource-rich areas now opt to use VL from the start to secure NTI rather than DL [30]. When attempting NTI with a VL, the use of SUZY/curved vascular forceps may be more effective (rather than Magill forceps) due to the increased curvature that better suits the hyperacute angle of VL blades, and maintaining a neutral head position has also been found to be superior to the sniffing position [31,32]. In addition, cuff inflation and alignment techniques can also be employed together with VLs to help secure NTI [33].

Several recent case reports describe successful utilization of both FIS and VL during difficult NTI (hybrid technique) [34,35,36]. This hybrid technique is especially helpful following failed intubation attempts with FIS or VL alone. A variety of devices other than VLs and FIS have also been used to facilitate NTI when confronted with a difficult CL grade view 3/4. Among these, lighted stylet devices utilizing the transillumination technique, such as the lightwand (Trachlight) and intubating lighted video stylets, such as Trachway and Disposcope endoscope, are prominent [37,38,39].

### 2.2. Awake NTI Approach

When faced with a patient who may be potentially difficult to ventilate and/or intubate, clinicians may opt to perform an awake NTI if the patient is cooperative. One of the old techniques that is available to clinicians is to place a nasotracheal tube blindly (BNTI). Fritz Kuhn had used BNTI as early as 1902. BNTI can be performed either in an awake, cooperative patient with judicious sedation and airway topicalization or attempted asleep in a child or uncooperative patient while maintaining spontaneous respiration. The patient is usually positioned with the neck flexed and head extended at the atlantoaxial joint (“sniffing position”), and the ETT is directed posteriorly along the nasal floor into the hypopharynx and advanced gently towards the glottis, taking care to stay in the midline. Listening to the air flow with breaths helps to advance the NETT. Once at the laryngeal inlet, the tube is advanced into the trachea as the patient is asked to take a deep inspiration to abduct the vocal cords [40,41]. (Video S2) https://emedicine.medscape.com/article/1663655-technique?form=fpf#showall (accessed on 1 October 2025). Over time, investigators have modified the original BNTI that “went by the ear” to make it “less blind” by adding features, such as Beck Airway Airflow Monitor (BAAM) whistle, directional tip Endotrol tubes (Mallinckrodt, Hazelwood, MO, USA), cuff inflation, lighted stylets, video stylets, and ETCO2 monitoring [42,43,44].

The more popular approach for awake NTI is to use a FIS to guide the NETT into the trachea [45]. Adequate topical or regional anesthesia of the airway generally determines the ease and comfort of awake fiberoptic intubation [46]. Directional tip tubes, such as an EndoFlex tube, can assist FIS to guide it towards the glottis, and a 90-degree anticlockwise pre-rotation of the standard nasal RAE tube can help with a higher initial rate of successful railroading [47,48,49]. Alternatively, clinicians can consider initial placement of a spirally split nasopharyngeal airway (SNPA) as a conduit for easier passage of the FIS through the nasopharynx and without triggering much coughing and discomfort for the awake patient [50]. Judicious conscious sedation is also essential to reduce patient anxiety, enhance cooperativeness, and decrease hemodynamic disturbances. Benzodiazepines, propofol, opioids, alpha2-adrenergic agonists such as dexmedetomidine, and ketamine are the main classes of drugs that have been described to facilitate awake NTI [51].

Just as in the RNTI approach, other devices, such as VLs and lighted video stylets and techniques such as cuff inflation and hybrid VL and FIS, can also be used to assist the awake NTI approach [52,53]. These devices and techniques are particularly handy for awake NTI when secretions and epistaxis make glottic visualization with a FIS indiscernible or when there is difficulty advancing a NETT over the FIS.

Another awake NTI approach available for difficult airways and/or maxillofacial trauma is the retrograde nasal intubation technique. It involves the insertion of a guide wire through the cricothyroid membrane, which is then passed upward through the pharynx and delivered out of a nostril in a retrograde manner. A guiding Cook catheter followed by a NETT is then advanced into the trachea using the wire as a guide [54,55].

### 2.3. Initial Orotracheal Intubation (OTI) Approach

An initial OTI has several situational advantages: (1) By securing ventilation and oxygenation, it can abate the need for intermittent mask ventilation, which may occasionally be difficult during the RNTI approach or preferentially avoided with complicated mandibular fractures. (2) Since OTI can be accomplished quicker and easier than a NTI, this approach is especially useful in high aspiration risk situations by providing clinicians a chance to protect the airway and decompress the stomach with a nasogastric tube (NGT) before conversion to NTI is attempted. (3) The initial OTI approach and conversion to NTI is also a favorable option when the patient arrives in the operating room already orally intubated from the field, emergency room, or ICU. (4) Securing the airway orally allows ample time to employ additional epistaxis reduction measures (if desired) prior to placement of the NETT into the nasopharynx and protects the airway from aspiration of heme if severe epistaxis occurs. (5) OTI can also be used as a backup option when difficulty is encountered during RNTI, as noted by Backman et al. [6].

## 3. Orotracheal Intubation (OTI) to Nasotracheal Intubation (NTI) Conversion

**Figure 2:**
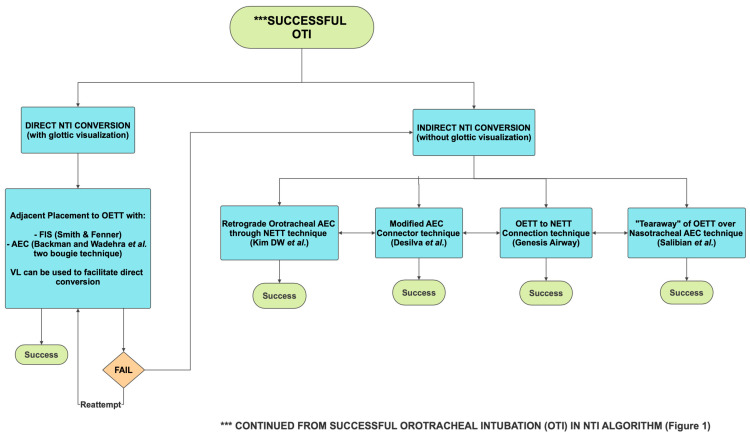
Orotracheal Intubation (OTI) to Nasotracheal Intubation (NTI) conversion algorithm illustrates published OTI to NTI conversion techniques to date, including direct (with glottic visualization) and indirect (without glottic visualization) pathways [6,56,57,58,59,60,61]. Reprinted with permission. NTI = Nasotracheal Intubation; OTI = Orotacheal Intubation; OETT = Oral Endotracheal Tube; NETT = Nasal Endotracheal Tube; FIS = Flexible Intubation Scope; AEC = Airway Exchange Catheter; VL = Videolaryngoscope; NETT = Nasal Endotracheal Tube.

Successful conversion from OTI to NTI can be accomplished through a variety of techniques, offering the clinician options suited to their preference and available equipment (Figure 2).

Once OTI is secured and confirmed, the clinician may proceed by two primary pathways:

### 3.1. Direct OTI to NTI Conversion with Glottic Visualization

A major benefit of direct OTI to NTI conversion techniques is the continuation of ventilation and oxygenation up until the final step of advancing the NETT over a FIS or Airway Exchange Catheter (AEC). Such a reduction in apnea time is a very appealing feature, especially for less experienced clinicians.

The Smith & Fenner FIS Guided direct conversion technique advances a FIS (loaded with a NETT) through the nasal passage and oropharynx and then guides it through the vocal cords adjacent to the existing OETT. With slight deflation of the OETT cuff, the FIS is advanced into the trachea until the carina is visualized. The OETT is then removed (either completely or over an AEC) and the NETT is advanced over the FIS into the trachea [56]. (Video S3) https://m.youtube.com/watch?v=z1hhHxMaAD0 (accessed on 1 October 2025).

The Backman & Wadhera “Two Bougie” direct conversion technique leverages two bougies (AECs) for continuous airway security. Initially, one bougie (AEC) is inserted nasally and guided into the trachea alongside the OETT under DL glottic visualization. The OETT is then removed over another bougie (AEC), leaving two bougies (AECs) in the airway transiently. The NETT is then threaded over the nasal bougie, and ETCO2 is confirmed to complete the NTI. The oral bougie (AEC) is subsequently removed but offers a guide to reinsert the OETT in case any unanticipated difficulties arise during advancement of the NETT into the trachea [6,57]. (Video S4) https://m.youtube.com/watch?v=9KgkQ7R9BB4 (accessed on 1 October 2025).

Challenges to direct conversion techniques may occur due to blood and secretions in the airway, obscuring the FIS view, or from a poor CL glottic view during DL. However, it can often be circumvented by using a VL to obtain a better view of the glottis during the OETT to NETT exchange [62,63]. Another consideration to improve the glottic view during these direct OTI to NTI conversion techniques is the posterior displacement of the OETT manually from outside the mouth towards the palate, which often drags the larynx and vocal cords into view during direct or video laryngoscopy [64].

### 3.2. Indirect OTI to NTI Conversion Without Glottic Visualization

Indirect nasotracheal conversion, which avoids direct visualization of the glottis during the OTI to NTI conversion, offers flexibility when equipment inaccessibility or challenging direct conversion attempts are encountered. Five innovative indirect OTI to NTI conversion techniques have been described in the literature:

The Retrograde AEC indirect conversion technique described by Kim DW et al. [58] begins with an initial OTI after which a NETT together with an AEC is inserted into the oropharynx through a naris. The AEC and NETT are then retrieved outside the mouth from the oropharynx with Magill forceps. With the tip of the NETT now outside the mouth, the AEC is fully withdrawn from the NETT. The AEC is reinserted through the OETT, and the OETT is extubated, leaving the AEC in the trachea. The proximal end of the oral AEC is then inserted retrograde through the tip of the NETT, which was pulled out of the mouth earlier. Finally, the NETT is railroaded or tracked over the nasotracheal AEC into the trachea, securing the NTI conversion [58]. (Video S5) https://m.youtube.com/watch?v=fz3REayMf8M (accessed on 1 October 2025).

The Modified Connector indirect conversion technique by Desilva et al. [59] is an innovative technique that revitalizes the original connector technique by Nakata & Niimi, utilizing a Patil Connector [65]. After initial OTI, an 18 Fr NGT is inserted nasally and retrieved out of the mouth manually or using Magill forceps. Following a cut and slit modification of the NGT’s distal end, a 14 Fr AEC is advanced through the OETT, and the OETT is extubated, leaving the 14 Fr AEC in the trachea. The proximal end of the AEC is seated into the slit distal end of the NGT outside of the mouth, and two silk sutures are placed through the walls of both the NGT and AEC to secure the connection. The NGT-AEC connection is then withdrawn nasally (being cautious not to extubate the distal end of the 14 Fr AEC from the trachea), establishing a nasotracheal AEC conduit to guide advancement of a NETT into the trachea [59]. (Video S6) https://m.youtube.com/watch?v=on9TgMstpsk (accessed on 1 October 2025).

The Tear-Away indirect conversion technique by Salibian et al. [60] involves initial OTI followed by insertion of a pediatric Cook AEC nasally until visualized in the oropharynx and retrieving the distal end of the AEC out of the mouth. After the OETT connector is removed, the AEC is inserted through the OETT into the trachea. The OETT without the connector is then cut longitudinally with a 10-blade in a “tear-away” fashion as it is withdrawn over the AEC. This preserves the nasotracheal AEC conduit in place to advance a NETT into the trachea to complete the NTI conversion [60]. (Video S7) https://m.youtube.com/watch?v=_amqGHno5GI (accessed on 1 October 2025).

The Genesis Interchangeable Oral-Nasal ETT Connection indirect conversion technique offers a reintubation-free transition, unlike the other three indirect conversion options described above. The Genesis Airway ETT (Genesis Airway, Sunshine Coast, Australia) employs a reinforced endotracheal tube (RET) with a centered, posterior-facing bevel and a curved tip similar to the Parker Flex-Tip tube (Parker Medical, Highlands Ranch, CO, USA) [61]. The Parker Flex-Tip tube has been shown to reduce trauma and hang-up during NETT insertion and advancement compared to a standard ETT [66,67].

Starting with an OTI using the distal RET part of the Genesis ONETT Airway, the proximal flexible part of the Genesis ONETT Airway, connected to a soft curved obturating introducer, is then inserted nasally into the oropharynx and pulled out of the mouth with Magill forceps. The ends of the flexible proximal part (after removal of the introducer) and distal RET part (after removal of the ETT connector) of the Genesis ONETT Airway have been manufactured to easily connect together firmly, allowing for a continuous NETT. The NETT can then be slowly withdrawn nasally until the proximal flexible part can be disconnected, leaving behind the proximal end of the distal RET outside the nose, securing the NTI conversion [68]. (Video S8) https://m.youtube.com/watch?v=LZ7XWWKS8yU (accessed on 1 October 2025).

The downside of the Genesis technique is the rare availability of the Genesis Interchangeable Oral Nasal intubation kit in the operating room, and also the fact that the pilot cuff of the NETT is left out of the mouth (as opposed to out of the nose, away from the surgical field with the other indirect conversion techniques). The surgery team will have to either sink the pilot cuff in the oropharynx or maneuver the pilot cuff around as needed during surgery to avoid cuff damage.

Retrograde oral to nasal indirect conversion techniques have also been published in the literature but predominantly in pediatric patients [69,70]. The smaller sizes of ETT employed in pediatric patients make retrograding feasible through the narrow, rigid nasal airway opening at the choanae. This approach is not so favorable in adults due to potential trauma to nasopharyngeal structures, such as the Eustachian tube orifice, from retrograding larger-sized ETTs.

An important caution to mention when using AECs in these conversion methods is to avoid inserting the AEC too far past the carina and potentially causing bronchial trauma. This can be avoided by paying attention to the markings on the AEC and making sure the AEC does not go past the 20–25 cm marking beyond the incisors of the mouth or past the 27–30 cm marking at the nares, depending on the height of the adult patient [71].

## 4. Controlling Epistaxis

Epistaxis is a common complication during NTI due to the rich vascularity and friability of the nasal mucosa. Minimizing epistaxis improves visualization and enhances safety by allowing for more options to secure NTI and reduce possible aspiration of blood. Although the severity of epistaxis during NTI is usually mild and transient, at times it can be severe [11,72]. So, it is paramount that clinicians consider interventions to reduce this complication when NTI is attempted.

Foremost, it is essential to recognize risk factors for severe epistaxis, such as a history of frequent epistaxis, anticoagulant use, or bleeding disorders, in addition to other absolute contraindications for NTI [73]. For these patients, the deficient clotting factors or platelets must first be replenished, the anticoagulant must be stopped for an adequate time, and/or alternative airway options should be considered.

Nasal flow rate assessment, preoperative bilateral nasal fiberoptic endoscopy exam by an experienced otolaryngologist (ENT) or anesthesia provider, and preoperative evaluation of paranasal sinuses on head Computed Tomography (CT)/skull radiograph imaging are helpful in identifying significant asymptomatic intranasal abnormalities such as bony spurs/septal deviations that impact patency and choice of nostril for NTI [74,75,76]. The right nostril appears to be generally more appropriate for NTI when both nostrils are symmetrically patent, based on lower incidence/severity of epistaxis and faster intubation time but similar nasal passage time [77,78,79,80]. Keeping the bevel tip of a NETT on the lateral side of the nostril or facing cephalad appears to help reduce epistaxis and the risk of middle turbinectomy [81,82,83].

Clinicians have a slew of epistaxis-reducing interventions to choose from. The type of endotracheal tube matters based on its composition and design, the design of the tube tip, and its softness [84,85]. “Telescoping the NETT” with a red rubber Robinson/Jaques Nelaton catheter or “obturating the NETT” with a NGT/an esophageal stethoscope is conducive for safe navigational guidance through the nasal pathway and significantly decreases the incidence and severity of epistaxis during NTI [86,87,88,89,90]. (Supplementary S1). Plus, if the red rubber Robinson catheter or obturator (NGT/esophageal stethoscope) is confirmed in the oropharynx prior to advancing the NETT into the nasopharynx, it will help prevent major unintended perforation injuries that can potentially occur from the NETT [91,92,93,94]. Another additional benefit of the red rubber catheter guided technique is the reduction in NETT tip contamination by nasal mucosa, decreasing transfer of tissue/bacteria from the nasal cavity into the lungs [95].

Using the lower nasal pathway between the inferior turbinate and nasal floor is safer in comparison to the upper nasal pathway because the lower pathway is away from the middle turbinate and cribriform plate [7,96,97]. Both nasal tip lifting and use of a red rubber catheter or NGT as a guide during NETT insertion help facilitate passage through the lower nasal pathway, reducing the incidence and severity of epistaxis [98].

Preparation of the nasal passage is useful to reduce epistaxis. Using vasoconstrictive agents such as oxymetazoline or phenylephrine can reduce vascular engorgement, while lubrication with lidocaine jelly provides both lubrication and local anesthesia to minimize sympathetic response [99]. Progressive dilation of the naris with nasopharyngeal airways of increasing size can help dilate the nasal tissues and reduce the risk of mucosal damage, albeit with variable efficacy [100,101,102]. Thermosoftening NETT with warm saline (~35 °C) and controlling the hypertension induced by the autonomic sympathetic response during NTI with sedatives, antihypertensives, and opioids can also help reduce the severity of epistaxis [103,104]. Finally, combining NETT telescoping/obturating together with thermosoftening has also been shown to be more efficacious than either technique alone [105].

## 5. Conclusions

Nasotracheal intubation (NTI) is a critical airway management technique. The decision-making process for NTI begins with the selection of an appropriate initial approach based on the clinical situation, likelihood of difficult ventilation or intubation pre-induction, and patient cooperativeness, as well as the clinician’s preference based on prior experience/comfort. Subsequent decision-making in the NTI pathway varies based on the initial approach chosen. Epistaxis reduction measures should always be applied to reduce the severity of this common complication and minimize its effect on NTI securement. By using the updated NTI Algorithm to guide them and having knowledge of the variety of techniques described in this manuscript, clinicians can not only start with a safe initial approach to secure NTI but also adapt to different clinical scenarios to optimize procedural success.

### Methodology

The changes to the foundational Piepho et al. and Backman et al. [5,6] flowchart and the choice of techniques are the opinions of the authors and based on their assessment of which techniques are best established in the literature, safe and meaningful to include. The authors believe the general outline holds true for most clinical situations. Of course based on the access to adjunct equipment and variable clinician experience, preference of approach/technique may change and some may bypass some steps and jump directly to the adjunct device or technique(s) of their choice.

## Supplementary Materials

The following supporting information can be downloaded at: https://www.mdpi.com/article/10.3390/jcm14217746/s1, Video S1: https://m.youtube.com/watch?v=RxWc8JK0_iE (accessed on 1 October 2025) (on page 1) illustrates the “routine Nasotracheal Intubation” (RNTI) approach, which is an asleep NTI with the help of direct laryngoscopy (DL) and Magill forceps. Video S2: https://emedicine.medscape.com/article/1663655-technique?form=fpf#showall (accessed on 1 October 2025) [41] illustrates the blind nasotracheal intubation technique courtesy of Therese Canares, MD, and Jonathan Valente, MD, Rhode Island Hospital, Brown University. Video S3: https://m.youtube.com/watch?v=z1hhHxMaAD0 (accessed on 1 October 2025) illustrates the Smith & Fenner FIS Guided direct OTI to NTI conversion technique, which advances an FIS (loaded with an NETT) through the vocal cords adjacent to the existing OETT. Video S4: https://m.youtube.com/watch?v=9KgkQ7R9BB4 (accessed on 1 October 2025) illustrates the Backman & Wadhera “Two Bougie” direct OTI to NTI conversion technique, which leverages two bougies (AECs) for continuous airway security. Video S5: https://m.youtube.com/watch?v=fz3REayMf8M (accessed on 1 October 2025) illustrates the “Retrograde AEC” indirect OTI to NTI conversion technique described by Kim DW et al. [58], where the proximal end of an oral AEC is inserted retrograde through the tip of a NETT withdrawn out of the mouth earlier. Then, the NETT is railroaded or tracked over the nasotracheal AEC into the trachea. Video S6: https://m.youtube.com/watch?v=on9TgMstpsk (accessed on 1 October 2025) illustrates the “Modified Connector” indirect OTI to NTI conversion technique by Desilva et al. [59], whereby the proximal end of an AEC is seated into the slit distal end of a NGT outside of the mouth and sutured together to establis a NGT-AEC connection and a nasotracheal AEC conduit to guide advancement of a NETT into the trachea; Video S7: https://m.youtube.com/watch?v=_amqGHno5GI (accessed on 1 October 2025) illustrates the “Tear-Away” indirect OTI to NTI conversion technique by Salibian et al. [60] whereby a nasal AEC is withdrawn out of the mouth and inserted through an OETT (after removal of the OETT connector) into the trachea. The OETT without the connector is then cut longitudinally in a “tear-away” fashion, preserving the nasotracheal AEC conduit in place to advance an NETT into the trachea. Video S8: https://m.youtube.com/watch?v=LZ7XWWKS8yU (accessed on 1 October 2025) illustrates the “Genesis Interchangeable Oral-Nasal ETT Connection” indirect OTI to NTI conversion technique, where the distal end of an NETT (after removal of the introducer) and proximal OETT (after removal of the OETT connector) of the Genesis ONETT Airway have been manufactured to easily connect together firmly, allowing for a continuous NETT. Supplementary S1: https://doi.org/10.4236/ojanes.2017.78028 [12] illustrates the red rubber catheter-guided technique for NTI and cites evidence for significant epistaxis reduction with this method.
