# Supplementary material for: Refining the Clinical Pathway for Nasotracheal Intubation: An Updated Decision Making Algorithm
Source: J Clin Med. 2025 Oct 31;14(21):7746. doi: 10.3390/jcm14217746 (PMC12610429; doi:10.3390/jcm14217746)
Supplement: Supplementary file 1 [file jcm-14-07746-s001.zip › jcm-3920296-supplementary.pdf]

SUPPLEMENTARY DIGITAL CONTENT:

VIDEO S1

https://m.youtube.com/watch?v=RxWc8JK0_iE (on page 1) illustrates the **"routine Nasotracheal Intubation"(RNTI) approach** which is an asleep NTI with the help of direct laryngoscopy (DL) and Magill forceps

VIDEO S2

https://emedicine.medscape.com/article/1663655-technique?form=fpf#showall [41] illustrates the blind nasotracheal intubation technique courtesy of Therese Canares, MD, and Jonathan Valente, MD, Rhode Island Hospital, Brown University.

VIDEO S3

https://m.youtube.com/watch?v=z1hhHxMaAD0 illustrates the **Smith & Fenner FIS Guided direct OTI to NTI conversion technique** which advances a FIS (loaded with a NETT) through the vocal cords adjacent to the existing OETT.

VIDEO S4

https://m.youtube.com/watch?v=9KgkQ7R9BB4 illustrates the **Uria & Wadhera "Two Bougie" direct OTI to NTI conversion technique** which leverages two bougies (AECs) for continuous airway security.

VIDEO S5

https://m.youtube.com/watch?v=fz3REayMf8M illustrates the "**Retrograde AEC" indirect OTI to NTI conversion technique described by Kim DW et al.**, where the proximal end of an oral AEC is inserted retrograde through the tip of a NETT withdrawn out of the mouth earlier. Then the NETT is railroaded or tracked over the nasotracheal AEC into the trachea.

VIDEO S6

https://m.youtube.com/watch?v=on9TgMstpsk illustrates the "**Modified Connector" indirect OTI to NTI conversion technique by Desilva et al.**, whereby the proximal end of an AEC is seated into the slit distal end of a NGT outside of the mouth and sutured together to establis a NGT-AEC connection and a nasotracheal AEC conduit to guide advancement of a NETT into the trachea

VIDEO S7

https://m.youtube.com/watch?v=_amqGHno5GI illustrates the "**Tear-Away" indirect OTI to NTI conversion technique by Salibian et al**. whereby a nasal AEC is withdrawn out of the mouth and inserted through an OETT (after removal of the OETT connector) into the trachea. The OETT without the connector is then cut longitudinally in a tear-away" fashion preserving the nasotracheal AEC conduit in place to advance a NETT into the trachea.

VIDEO S8

https://m.youtube.com/watch?v=LZ7XWWKS8yU illustrates the "**Genesis Interchangeable Oral-Nasal ETT Connection" indirect OTI to NTI conversion technique,** where the distal end of a NETT (after removal of the introducer) and proximal OETT (after removal of the OETT connector) of the Genesis ONETT Airway have been manufactured to easily connect together firmly allowing for a continuous NETT.

SUPPLEMENTARY S1

https://doi.org/10.4236/ojanes.2017.78028 [12] illustrates the red rubber catheterguided technique for NTI and cites evidence for significant epistaxis reduction with this method.
